# Comparison of initial learning algorithms for long short-term memory method on real-time respiratory signal prediction

**DOI:** 10.3389/fonc.2023.1101225

**Published:** 2023-01-20

**Authors:** Wenzheng Sun, Jun Dang, Lei Zhang, Qichun Wei

**Affiliations:** ^1^ Department of Radiation Oncology, The Second Affiliated Hospital, School of Medicine, Zhejiang University, Hangzhou, Zhejiang, China; ^2^ Department of Radiation Oncology, National Cancer Center/National Clinical Research Center for Cancer/Cancer Hospital and Shenzhen Hospital, Chinese Academy of Medical Science and Peking Union Medical College, Shenzhen, Guangdong, China; ^3^ Department of Oncology, The First Affiliated Hospital of Chongqing Medical University, Chongqing, China; ^4^ Graduate Program of Medical Physics and Data Science Research Center, Duke Kunshan University, Kunshan, Jiangsu, China

**Keywords:** respiratory signals prediction, initializer, long short-term memory, radiation therapy, He initializer, Glorot initializer, orthogonal initializer, narrow-normal initializer

## Abstract

**Aim:**

This study aimed to examine the effect of the weight initializers on the respiratory signal prediction performance using the long short-term memory (LSTM) model.

**Methods:**

Respiratory signals collected with the CyberKnife Synchrony device during 304 breathing motion traces were used in this study. The effectiveness of four weight initializers (Glorot, He, Orthogonal, and Narrow-normal) on the prediction performance of the LSTM model was investigated. The prediction performance was evaluated by the normalized root mean square error (NRMSE) between the ground truth and predicted respiratory signal.

**Results:**

Among the four initializers, the He initializer showed the best performance. The mean NRMSE with 385-ms ahead time using the He initializer was superior by 7.5%, 8.3%, and 11.3% as compared to that using the Glorot, Orthogonal, and Narrow-normal initializer, respectively. The confidence interval of NRMSE using Glorot, He, Orthogonal, and Narrow-normal initializer were [0.099, 0.175], [0.097, 0.147], [0.101, 0.176], and [0.107, 0.178], respectively.

**Conclusions:**

The experiment results in this study indicated that He could be a valuable initializer in the LSTM model for the respiratory signal prediction.

## 1 Introduction

During radiation therapy treatment delivery process, tumor in certain organs, such as the lung, would be subject to substantial motion due to patient respiration (1–5). This motion may lead to the leakage of radiation dose from the tumor target to nearby normal tissues, which would sharply degrade the accuracy and quality of the radiation therapy treatment. Respiratory motion could be measured and monitored by several mature techniques. However, the real-time adaptation to motion during radiotherapy treatment is challenging, and latencies in hundreds of milliseconds may still exist (6–10). Hence, prediction of the tumor motion in advance could help reduce these latencies and improve the quality of radiotherapy treatment in mobile cancers.

Many machine learning models have been proposed to predict the respiratory motion. Putra et al. investigated the prediction performance of the Kalman filter (KF) for a short latency (11). Recent studies have demonstrated the merit of artificial neural network (ANN) models on respiratory signal prediction, especially for the nonlinear signals (3, 12, 13). Sharp et al. indicated that the ANN models could have better prediction performance as compared to the KF method (6). Sun et al. proposed a revamped multilayer perceptron neural network (MLP-NN) called Adaboost MLP-NN (ADMLP-NN), which showed more accurate predictions than the MLP-NN (3). One of the main limitations of the ANN models was that they generally ignore the temporal dependence of the previous inputs. The recurrent neural network (RNN) was introduced to include the consideration of temporal information. However, the gradient disappearance and explosion problems restricted the application of RNN on long-term memory prediction. A special RNN model known as long short-term memory (LSTM) (14) had been proposed to overcome the above weakness of RNN (gradient disappearance and explosion problems). Various studies have demonstrated the superior performance of the LSTM model in different time-series prediction tasks (15–20), including the respiratory signal prediction. Wang et al. showed that the prediction performance of a suitable LSTM model could be three-fold higher than that of the ADMLP-NN model (18). Another two studies also showed the potential of the LSTM model in the respiratory signal prediction (19, 20).

Neural network models are sensitive to their initial weights (21, 22). When the neural network was successfully proposed initially, the Narrow-normal initializer was usually used to generate the initial value of weight from a predefined normal sampling distribution. The weights between layers are initialized using a fixed variance distribution, which may cause the problem of gradient disappearance or gradient explosion (21, 22). This defect hampered the extensive use of the Narrow-normal initializer. The Glorot initializer (23) provided a normalized initialization, which could maintain the activation and the back-propagated gradient variances during training based on the linear activation. Then, He et al. took the rectifier nonlinearity into account and proposed the He initializer (24). Sachs et al. illustrated that if the initial weight obeyed an orthogonal matrix, the initial conditions could keep the error vector norm through the deep neural network during back propagation process while generating depth independent learning times. Based on this finding, the orthogonal initializer (25) was proposed.

While the critical importance of the proper weight initializers on time-series prediction performance has often been noted in previous works (21–26), researchers often focused on improving the model architecture. Therefore, there is a lack of knowledge on the effect of initializer on respiratory signal prediction using the LSTM model. The aim of this study was to examine the effect of different initializer on the performance of LSTM model in the patient respiratory signal prediction. The primary contributions of this study were concluded as follows:

1. To the best of our knowledge, this is the first study to investigate the effectiveness of the weight initializers on the respiratory prediction problem using the LSTM model. In this study, we investigated the influence of the four common weight initializers discussed in the literature (22, 27) on the prediction performance of LSTM model using 304 breathing motion cases from an open-access database collected by the CyberKnife Synchrony tracking system (Accuray, Sunnyvale, CA) with a 26-Hz sampling rate.

2. We further investigated the effect of the irregular breathing patterns on the prediction performance for each initializer and demonstrated the advantage of using the He initializer on irregular respiratory pattern patients.

The results illustrated that the initial weight algorithms in the LSTM model would exert substantial effect on the respiratory signal prediction performance. The He initializer could be an optimal choice for the respiratory signal prediction, especially for the irregular respiratory pattern patients.

## 2 Methods

### 2.1 Prediction process

**Figure 1:**
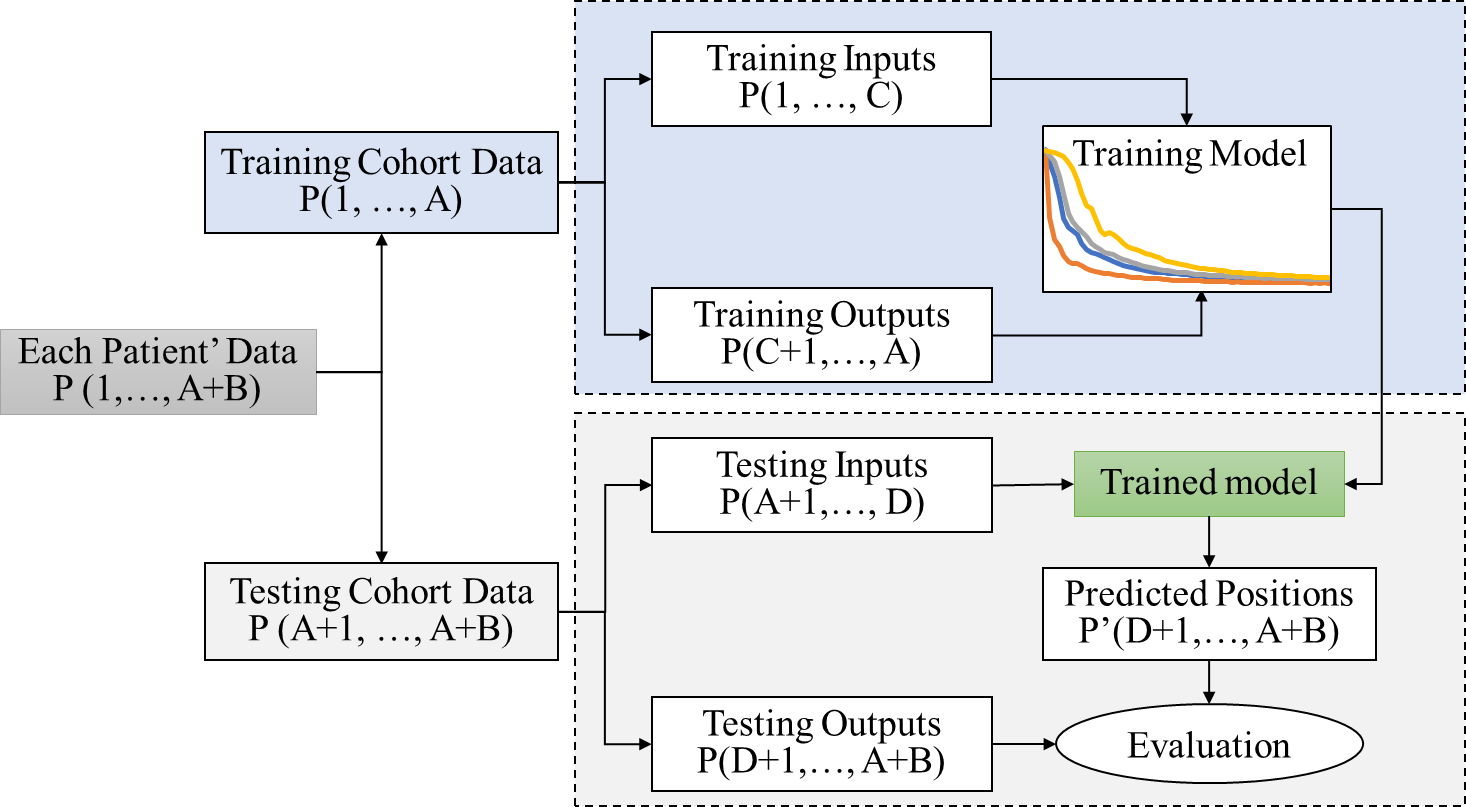
Flow chart of the prediction algorithm.

The general workflow for the prediction process used in this study is outlined in **Figure 1**. Each respiratory signal was divided into two segments separated by time A. Respiratory signals prior to time A were used as training data, and those after time A were used for testing. Among the training data, the signals before and after time C were used as the input and prediction outputs, respectively. LSTM neural network was implemented as the prediction model. The testing data (positions after the time A) were used to evaluate the developed LSTM prediction model. The testing data were divided into two segments separated by time D. The signals before time D were defined as the testing input, and those after time D were defined as the testing outputs or ground truth. The trained LSTM prediction model was applied to the testing inputs to generate the prediction signal P’, which was compared to the testing outputs or ground truth P for evaluation.

### 2.2 Long short-term memory neural network

A bidirectional architecture LSTM layer was developed to test the performance of all the four initializers in this study (17, 18). The formulas of the LSTM layer are illustrated in the **Equations 1–8**.


(1) $$i_{t}=\sigma \left(W_{xi}x_{t}\;+\;W_{hi}h_{t-1}\;+W_{ci}c_{t-1}\;+\;b_{i}\right)$$

(2) $$f_{t}=\sigma \left(W_{xf}x_{t}+W_{hf}h_{t-1}+W_{cf}c_{t-1}+b_{f}\right)$$

(3) $$c_{t}=f_{t}c_{t-1}+i_{t}tanh\left(W_{xc}x_{t}+W_{hc}h_{t-1}+b_{c}\right)$$

(4) $$o_{t}=\sigma \left(W_{xo}x_{t}+W_{ho}h_{t-1}+W_{co}c_{t}+b_{o}\right)$$

(5) $$h_{t}=o_{t}tanh(c_{t})$$

(6) $$h_{t}^{f}=tanh\left(W_{xh}^{f}x_{t}+W_{xh}^{f}h_{t-1}^{f}+b_{h}^{f}\right)$$

(7) $$h_{t}^{b}=tanh\left(W_{xh}^{b}x_{t}+W_{hh}^{b}h_{t+1}^{b}+b_{h}^{b}\right)$$

(8) $$y_{t}=W_{hy}^{f}h_{t}^{f}+W_{hy}^{b}h_{t}^{b}+b_{y}$$

Here, *i_t_*, *f_t_*, *c_t_*, *o_t_*, *h_t_*, $h_{t}^{f}$ , $h_{t}^{b}$ , and *y_t_* refer to the input gate, forget gate, memory cell vectors, output gate, hidden vector sequence, forward hidden vector sequence, backward hidden vector sequence, and output, respectively. The tanh and σ stand for the two activation functions as given by the **Equations 9** and **10**.


(9) $$\sigma (x)=\frac{1}{1\;+\;e^{-x}}$$

(10) $$\tanh(x)=\frac{e^{x}-e^{-x}}{e^{x}+e^{-x}}$$

***W*** *_xi_*, ***W*** *_hi_*, ***W*** *_ci_*, ***W*** *_xf_*, ***W*** *_hf_*, ***W*** *_cf_*, ***W*** *_xc_*, ***W*** *_hc_*, ***W*** *_xo_*, ***W*** *_ho_*, and ***W*** *_co_* denote the weighted parameters, while the ***b*** *_i_*, ***b*** *_f_*, ***b*** *_c_*, and ***b*** *_o_* represent the intercepts. A plurality of LSTM layers can be stacked into a deeper neural network, which can fit the complicated functions between the inputs and targets.

### 2.3 Initializers

For the Glorot initializers (also called the Xavier initializer), the weights *W_ij_* of each layer were initialized with the heuristic as follows (23):


(11) $$W_{i\;j}\;\sim \;U\left[-\sqrt{\frac{6}{N_{ℐ}+N_{O}}},\sqrt{\frac{6}{N_{ℐ}+N_{O}}}\right]$$

Here, *U*[ −δ, δ ] was sampled independently from a zero-mean uniform distribution in the bounds [ −δ, δ ] . *N_0_* was four times of the hidden unit number, and *N_J_* was the input channel number.

For the He initializer (24), the weights *W_ij_* were sampled according to a zero-mean normal (Gaussian) distribution with the following standard deviation (SD):


(12) $$W_{ij}\;\sim \;G\left[-\sqrt{\frac{2}{N_{ℐ}}},\sqrt{\frac{2}{N_{ℐ}}}\right]$$

Here, the size of *N_J_* was the input channel number for the input weight and the hidden unit number for the recurrent weight.

For the Orthogonal initializer (25), the orthogonal matrix ***Q*** was first established by producing the Gaussian matrices randomly and then computed by the ***QR*** decomposition using the formula ***Z*** = ***QR***, where ***Z*** obeyed unit normal distribution.

For the Narrow-normal initializer, the weights were obtained by sampling from a normal distribution with 0 mean and 0.01 SD independently.

### 2.4 Prediction performance evaluation

A total of 304 breathing motion traces collected by the CyberKnife Synchrony (Accuray, Sunnyvale, CA) tracking system and procured from an open dataset (18, 28) were used in this study. The detail information of the dataset is illustrated in **Table 1**. The first 1-min signal was used to train the LSTM model with different initial methods, while the following 30 s was applied to evaluate the effectiveness of each initial method. The ahead time was about 385 ms (10 samples). All the evaluation metrics were based on the following default hyper-parameters in this study: three LSTM layers, 0.001 initial learning rate, 50 time lags, and 300 hidden units.

**Table 1:** Detail information of the dataset used in this study.

Items	Result
Sampling Rate	26 Hz
Collected Equipment	CyberKnife Synchrony
Treatment Place	Georgetown University Hospital
Trace Number	304
Treatment Fraction	102
Patient Number	31
Private Information	None
Respiratory Signal Record Way	Fiducial marker on patient’s chest
Datasets Range in Duration	80–158 min
Imaging Data	None

RMSE is one of the main metrics used for respiratory signal prediction evaluation (2–4, 8–11, 19, 20). However, the root mean square error (RMSE) was not a dimensionless and normalized metric (29), and it would not be suitable for comparing the performance of respiratory signal prediction across different patients before normalization (12, 18). Hence, in order to minimize the effect of signal amplitude on different cases, the NRMSE instead of the RMSE between the real and predicted signal were used to evaluate the predication performance for all initializers (29). The NRMSEs used in this study are illustrated by **Equations 13** and **14**.


(13) $$NRMSE=\frac{\sqrt{\frac{1}{t_{A+B}-t_{\text{D}+1}+1}\sum_{t=t_{\text{D}+1}}^{A+B}{\left(P^{\prime }\left(t\right)-P\left(t\right)\right)}^{2}}}{Range\left(P\left(t\right)\right)}$$

(14) Range(P(t))=Max(P(t))−Min(P(t)) tϵ(D+1,A+B)

Here, *P*(*t*) and *P'*(*t*) were the ground truth and predicted signal at the time fame *t*, respectively.

Breathing irregularity was examined to test prediction performance of the LSTM model using the four initializers on different breathing patterns. As illustrated in the **Equation 15**, the breathing irregularity was defined as the average of the standard deviation (SD) of the maximum (i.e., peak) and minimum (i.e., valley) amplitudes. Patients were split into two groups by the median value of the irregularity (*r*= 0.22).


(15) $$r=\frac{SD_{peaks}+SD_{valleys}}{2}$$

## 3 Results

**Figure 2:**
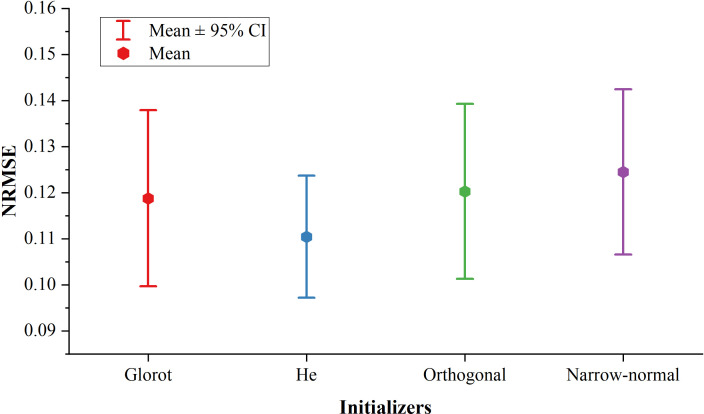
Prediction performance of the four initializers.

**Figure 2** shows the NRMSEs using four initializers (Glorot, He, Orthogonal, and Narrow-normal) with a 385-ms ahead time. The He initializer showed the best prediction performance. The mean of NRMSE using the He initializer was lower by 7.5%, 8.3%, and 11.3% compared to the Glorot, Orthogonal, and Narrow-normal initializer, respectively. The He initializer was more robust than the other three initializers, as it achieved the narrowest confidence interval (CI) among all the four initializers.

**Figure 3:**
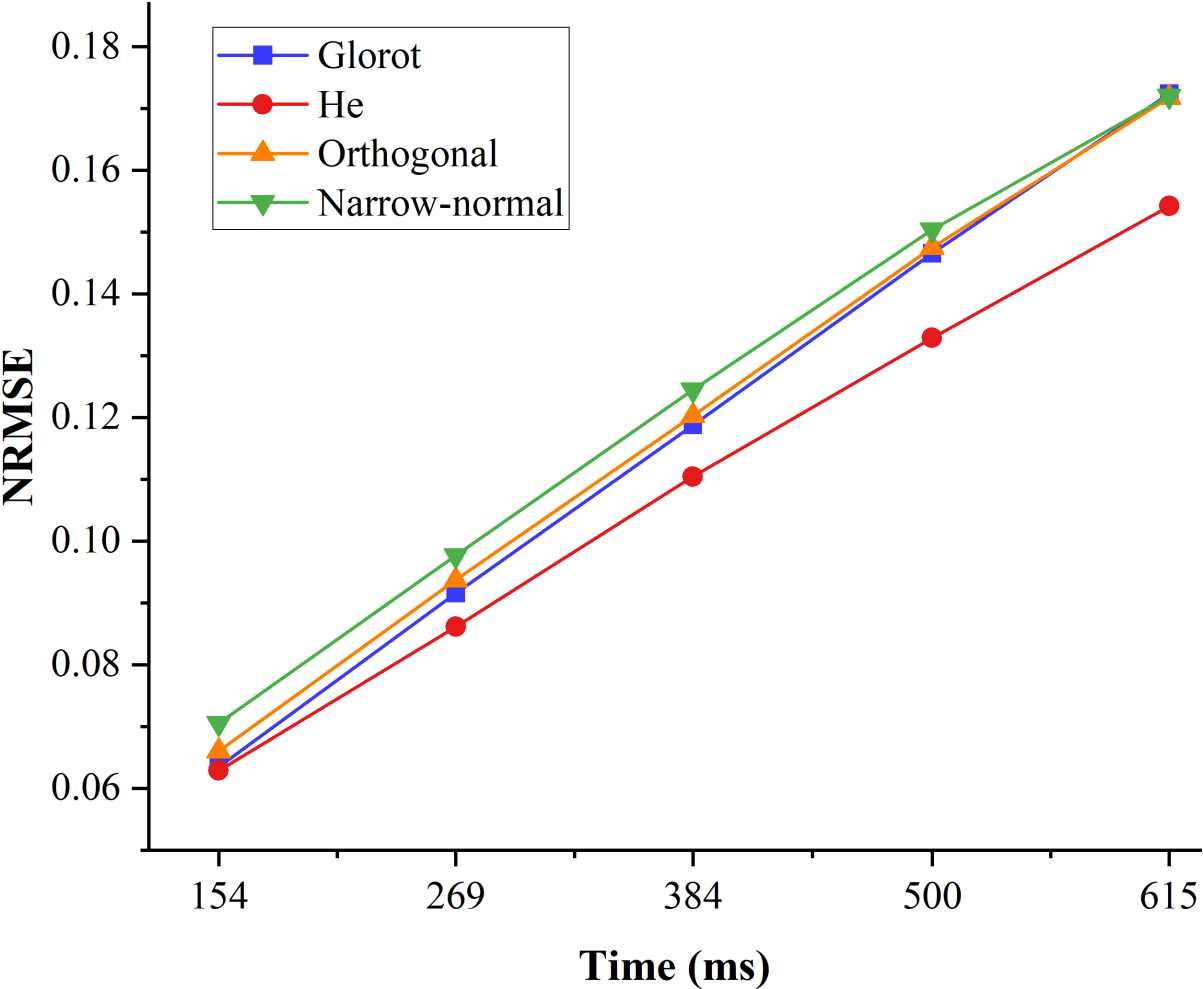
Prediction performance of the four initializers with different ahead times.

The prediction performance of the four initializers with different ahead time is shown in **Figure 3**. The prediction performance using all of the four initializers decreased when the ahead time increases. The prediction performance using the He initializer for all the ahead time examined in this study were higher than the other three initializers. The average prediction performance gap between the He initializer and other three initializers increased when the ahead time increases.

**Figure 4:**
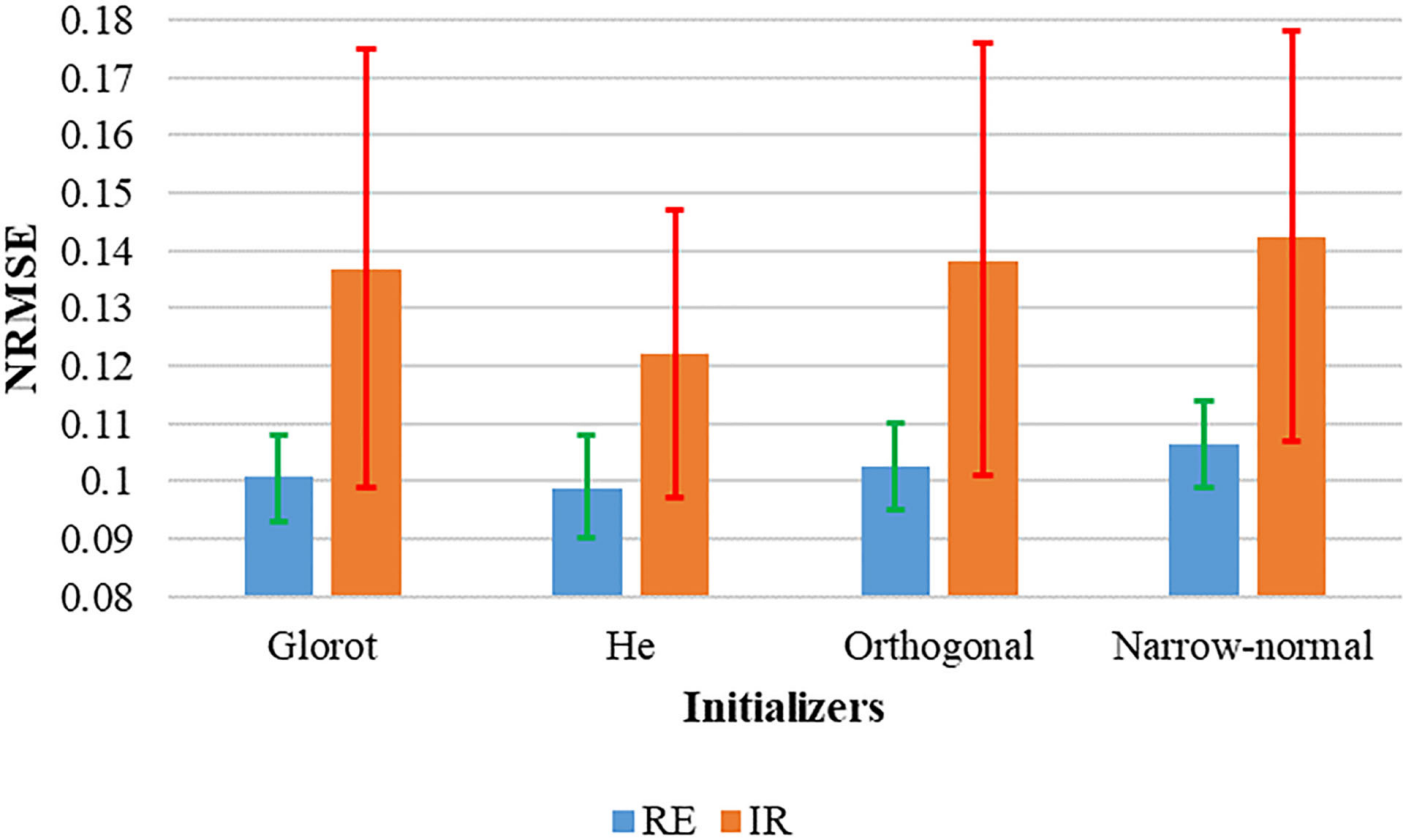
Prediction performance of the two groups divided by the median irregularity. The green and red bars represented 95% CI of the NRMSE for the RE and IR groups, respectively.

The prediction performance of the regular and irregular breathing groups, which are divided by the median irregularity is shown in **Figure 4**. For all the four initializers, the performance of the irregular group was inferior to the regular group. The prediction performances of Glorot, He, and Orthogonal were similar in the regular group. However, the mean of NRMSE in the irregular group using the He initializer was superior by 11.0% (Glorot), 11.6% (Orthogonal), and 14.1% (Narrow-normal), respectively. The upper limits of the 95% confidence interval (CI) of the NRMSE for the irregular group patients were lowest for He (0.147) and were 0.175, 0.176, and 0.178 for Glorot, Orthogonal, and Narrow-normal, respectively.

**Figure 5:**
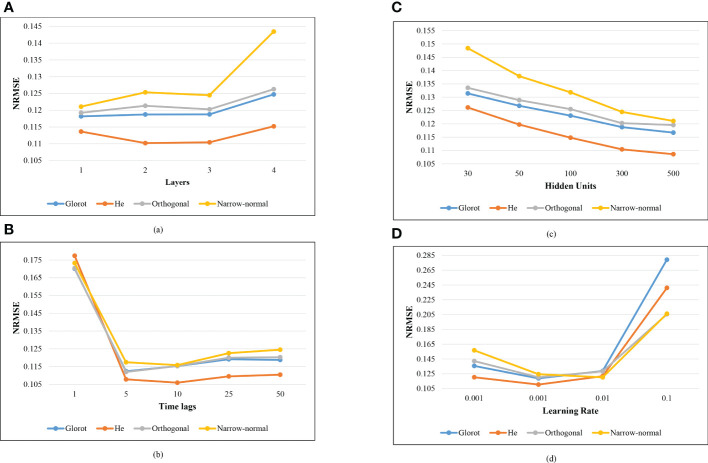
Effect of the hyper-parameters on the prediction performance. **(A)** Number of the LSTM layers (*N_l_*). **(B)** Time lags (*T_l_*). **(C)** Hidden units (*H_u_*). **(D)** Learning rate (*L_r_*).

The effect of the four important hyper-parameters on the prediction performance was explored and shown in **Figure 5**. Four different values (1, 2, 3, and 4) were selected to evaluate the effect of the ***N_l_*** on the prediction performance (**Figure 5A**). The LSTM model showed the similar prediction performance with one to three LSTM layers for the Glorot and Orthogonal initializers. For He and Narrow-normal initializers, the LSTM model with two or three LSTM layers showed similar prediction performance. The effect of ***T_l_*** on the prediction performance was examined by five selected values (1, 5, 10, 25 and 50) (**Figure 5B**). Time lag of 10 showed the best performance for the He and Narrow-normal initializers, while 5 showed the best performance for the other two initializers. The influence of ***H_u_*** was explored on five values (30, 50, 100, 300, and 500) (**Figure 5C**). The prediction performance kept improving as the ***H_u_*** increased from 30 to 500 for all the four initializers. Four ***L_r_*** values (0.0001, 0.001, 0.01, and 0.1) were investigated. The NRMSE using the first three initializers (Glorot, He, and Orthogonal) was lowest when ***L_r_*** was 0.001. Narrow-normal initializer showed the best prediction performance when ***L_r_*** was 0.01.

## 4 Discussion

The effect of four common initializers on the performance of respiratory signal prediction using the LSTM model was examined in this study. The results illustrated that the He initializer outperformed the other three initializers for its higher respiratory prediction performance. A suitable initializer would substantially improve the prediction performance.

The prediction performance using all the four initializers became lower when the ahead time increases. This was probably because the relationship between the training and predicted respiratory signals would diminish when the ahead time increases. However, the prediction performance deterioration using the He initializer was slower than the other three methods. This may suggest that the He initializer could enhance LSTM’s ability to capture longer ahead time information.

The prediction performance of the irregular breathing group was lower than the regular breathing group for all the four initializers. This may be contributed by the factor that the relationship between the prior and future signals in the irregular group was more difficult to capture than that in the regular group. The prediction performance of Glorot, He, and Orthogonal was similar in the regular group. However, the mean of NRMSE using the He initializer in the irregular group was lower compared to other three initializers. This suggested the superior ability of He initializer in capturing connection between prior and future signals. The upper limit of the 95% CI of the NRMSE using the He initializer was lower than that using other three initializers, suggesting that the He initializer might improve the general performance of the LSTM model in breathing signal prediction.

We also investigated the influence of hyper-parameters setting on the prediction performance for each initializer. A total of four important hyper-parameters was examined in this study. A large numerical value of the first three hyper-parameters (***N_l,_ T_l_***, and ***H_u_***) represented a complex network, which would fit a more complicated function but easy to overfit. The prediction performance became better as the first three hyper-parameters increased initially. However, as these three hyper-parameters continue to increase, the prediction performance improved only slightly and even could deteriorate. This may be because when these three hyper-parameters were too small, the LSTM model would not fit the respiratory curve well. Hence, the increase in these three hyper-parameters could improve the prediction performance initially. However, too large ***N_l_***, ***T_l_***, and ***H_u_*** would raise the risk of overfitting for the LSTM model and potentially degrade the prediction accuracy. The ***L_r_*** scales and updates the magnitude of the LSTM model weights to minimize the loss function. If ***L_r_*** was too small, the converge time would be long, and the risk of trapping in undesirable local minimum increases. On the other hand, if ***L_r_*** was too large, a suboptimal result may be obtained. Finally, 0.001 and 0.01 achieved the best performance for Narrow-normal and the other three initializers, respectively.

One of the limitations of this study was that all the respiratory signals from this database were originally detected by the fiducial marker placed on patient’s chest. These external signals may be different from the real internal tumor motion. Besides, the dataset was collected from a single center. In the future, we would further evaluate the effect of the initializers on actual tumor motion signals or internal respiratory signals ideally from multi-centers.

## 5 Conclusion

The influence of the four weight initializers on the performance of respiratory signal prediction using the LSTM model was investigated in this study. The results suggested that the weight initialization methods would exert substantial effect on the respiratory signal prediction performance. The He initializer could be an optimal initializer for the respiratory signal prediction using the LSTM model, especially for the irregular respiratory pattern patients.

## Data availability statement

The original contributions presented in the study are included in the article/supplementary material. Further inquiries can be directed to the corresponding author.

## Author contributions

WS and QW designed the methodology. WS and JD wrote the program. WS performed data analysis, interpretation and drafted the manuscript. LZ, JD, and QW revised this manuscript critically for important intellectual content and contributed to the interpretation of data. All authors reviewed the manuscript. All authors read and approved the final manuscript.
